# A population based survey in Ethiopia using questionnaire as proxy to estimate obstetric fistula prevalence: results from demographic and health survey

**DOI:** 10.1186/1742-4755-10-14

**Published:** 2013-02-25

**Authors:** Sibhatu Biadgilign, Yihunie Lakew, Ayalu A Reda, Kebede Deribe

**Affiliations:** 1 Department of Epidemiology and Biostatistics, College of Public Health and Medical Science, Jimma University, Jimma, Ethiopia; 2 Ethiopian Public Health Association (EPHA), Health and Demographic Surveillance Sites Coordinator for the EPHA-CDC project, Addis Ababa, Ethiopia; 3 Global Health, Brown Advanced Research Institutes (BIARI), Population Studies and Training Center (PSTC), Brown University, Providence, Rhode Island, USA; 4 Brighton and Sussex Medical School, Falmer, Brighton, UK

## Abstract

**Background:**

Obstetric Fistula (OF) remains a major public health problem in areas where unattended obstructed labor is common and maternal mortality is high. Obstetric Fistula was able to be prevented, treated and eradicated in high-income countries; however, it still affects many women in low-income countries. To our knowledge, only few studies have described the prevalence and factors associated with Obstetric Fistula in Ethiopia in population-based surveys.

**Objective:**

The aim of this study is to describe the prevalence and factors associated with Obstetric Fistula in Ethiopia.

**Methods:**

The study used women’s dataset from the 2005 Ethiopian Demographic and Health Survey. The survey sample was designed to provide national, urban/rural, and regional representative estimates of key health and demographic indicators. The sample was selected using a two-stage stratified sampling process. OF was measured using questionnaire. The data is analyzed using descriptive and multivariate statistical methods to determine factors associated with Obstetric Fistula.

**Results:**

A total of 14,070 women of reproductive age group were included in the survey. Of which 23.2% ever heard of obstetric fistula. Among women who ever given birth (9,713), some 103 (1.06%, 95% CI; 0.89%-1.31%) experienced obstetric fistula in their lifetime, which means 10.6 per 1000 women who ever gave birth. It is estimated that in Ethiopia nearly 142,387 (95% CI: 115,080-169,694) of obstetric fistula patients exist. Those women who are circumcised had higher odds of reporting the condition (Chi square = 4.41, p-value = 0.036). In the logistic regression model women from rural areas were less likely to report obstetric fistula than their urban counterparts (OR = 0.21, 95% CI: 0.06-0.69). Women who gave birth 10 or more had higher odds of developing obstetric fistula than women with 1-4 child (OR = 4.34; 95% CI; 1.29-14.55).

**Conclusions:**

Obstetric fistula is a major public and reproductive health concern in Ethiopia. This calls for increased access to emergency obstetric care, expansion of fistula repair service and active finding of women with OF with campaigns of ending fistula is recommended.

## Introduction

An estimated half a million women who die in pregnancy and childbirth each year, about 20 to 50% of them experience obstetric morbidities, which, if left untreated can cause lifelong pain and humiliation [1]. A further 20 million will experience pregnancy-related illnesses [2]. This is particularly the case for sub-Saharan Africa and South Asia, in which at least 87% of the estimated annual 342,900 maternal deaths worldwide occur according to recent estimates, with over 50% of all maternal deaths occurring in only six countries. The countries are India, Nigeria, Pakistan, Afghanistan, Ethiopia and the Democratic Republic of Congo [3]. The vast majority of maternal deaths are due to direct obstetrical complications, including hemorrhage, infection, eclampsia, obstructed labor, and unsafe abortion.

Obstetrical fistula, caused by prolonged obstructed labor, is a hole in the bladder or rectum opening into the vagina, through which urine or feces leak uncontrollably. At least 2 million women in developing countries are living with obstetrical fistulas, and 50,000 to 100,000 new cases occur each year, but these figures probably underestimate the problem [4]. A recent conservative attempt to estimate the incidence of obstetric fistulas with a population-based survey of severe obstetric morbidity in West Africa concluded that there were probably at least 33 000 new cases each year in sub-Saharan Africa [5]. In another study it was estimated that in sub-Saharan Africa alone, between 30,000 and 130,000 of women giving birth develop fistula each year [6]. Obstetric Fistula constitutes a serious threat to the reproductive health of adolescent women [7]. Obstetric Fistula was able to be prevented and treated in high-income countries; however, still it affects many women in low-income countries including Ethiopia. Fistulas occur in places where use and access to obstetric care is limited.

Although there were a paucity of sound data on the number of women living with fistula in Ethiopia [8], the 2005 Ethiopian Demographic and Health Survey attempted to examine the prevalence of the problem in the country. To our knowledge, no studies have described the prevalence and determinants of obstetric fistula in Ethiopia in population-based surveys including the 2011 Ethiopia Demographic and Health Survey. The aim of this study is to describe the prevalence and determinants of obstetric fistula in Ethiopia using a population-based cross-sectional design of the 2005 Ethiopian Demographic and Health Survey.

## Methods

### General description of the survey

This study is based on a secondary analysis of data from the 2005 Ethiopia National Demographic and Health Survey (EDHS). The survey was the second round in Ethiopia that fielded from April 27 to August 30, 2005. In the first round 15,365 women were interviewed while in the 2005 (second round) the survey was administered to 14,070 women aged 15-49 within 13,928 sampled households. Standardized questions developed by ORC Macro DHS for developing countries that were administered by the Ethiopian Central Statistical Agency. The survey questionnaire was pretested using major local languages. We used some specific questions that were included in the survey instrument and directly related towards OF such as have you ever heard of obstetric fistula (have you ever heard of a condition in which a woman continuously leaks urine and/or faces following childbirth, have you yourself experienced obstetric fistula and have you ever been treated for obstetric fistula? Responses were collected through face to face interview mode.

### Population and sampling

The sample was selected using a two-stage stratified sampling process. In the first stage, 540 clusters were selected from the list of enumeration areas from the Population and Housing Census. Fieldwork was successfully completed in 535 of the 540 clusters. In the second stage, 24 to 32 households were selected systematically from each cluster for the survey sample. In brief, the survey sample was intended to provide national, urban/rural, estimates of health and demographic parameters. The survey administered the Women’s Questionnaire to all eligible women age 15-49 in the sampled households.

### Measurements

The dependent variable for this study was women ever experienced obstetric fistula whereas the main independent variables were social-demographic, economic and maternal health care services that are expected to have association with obstetric fistula.

Operationally, we define the following terms. Circumcision – a circumcised woman is said to be reported that their vagina had been sewn closed (infibulations) and untreated fistula- those women who had developed OF but they are not getting any surgical interventions.

### Statistical analysis

The survey data were entered and processed using CSPro statistical software that was developed jointly by the U.S. Census Bureau, Macro International Inc., and Serpro, SA (Census and Survey Processing System, 2007). We analyzed the final data using STATA® version 10.0 for windows. To examine factors associated with OF; we used both the descriptive and multivariate statistical methods. Multivariable analysis was conducted using logistic regression models. In the descriptive analysis, the distribution of respondents by the key variables and the prevalence of OF was presented by the categories of each variable. In the multivariate or adjusted logistic regression models, the outcome variable (OF) was regressed on the selected predictor variables. The statistical tests were reported as significant if the level of confidence was 95 percent or greater. The prevalence data were entered using a Microsoft Excel 2007 spreadsheet and exported into ArcGIS to visualize key estimations. The regional prevalence and household prevalence were used to develop OF prevalence map using the ArcGIS® software.

### Ethical review

The study protocol was approved by the National Ethics Review Committee of the Ethiopia Science and Technology Commission in Addis Ababa, Ethiopia and the ORC Macro Institutional Review Board in Calverton, USA.

## Result

A total of 14070 women of reproductive age group were included in the survey. Of which 23.2% ever heard of OF. Among women who ever given birth (9,713) some 103 (1.1%) (10.6 per 1000 women who ever given birth) experienced obstetric fistula in their lifetime. Of the 103 cases 33 (32.0%) were ever treated, this gives the prevalence of untreated fistula to be 7.2 per 1000 women. Among women who ever heard about OF, 3.8% indicated other women in household with obstetric fistula. The mean age of women who reported OF was 32.9 (±9.6) (Table 1).

**Table 1:** Basic background information of participants

**Characteristics**	**Women experienced Fistula**		**Chi square**	**P-value**
**Region**	**Yes %(95% CI)**	**No (%)**		
Tigray	1.6(0.80, 2.40)	98.4	26.59	0.003
Afar	1.0(0.78, 1.78)	99.0		
Amhara	0.5(0.49, 0.51)	99.5		
Oromiya	1.2(0.66-1.74)	98.8		
Somali	0.0(0,0)	100		
Benishangul-Gumuz	0.6(0.56, 0.64)	99.4		
SNNPR	1.5(0.60,2.10)	98.5		
Gambela	1.1(0.85, 1.95)	98.9		
Harari	0.1(0.07,0.13)	99.9		
Addis Ababa	1.0(0.69,1.69)	99.0		
Dire Dawa	1.0(0.87,1.87)	99.0		
**Residence**			2.33	0.127
Urban	1.4(0.46,1.86)	98.6		
Rural	1.0(0.16, 1.16)	99.0		
**Age**			12.70	0.048
15-19	2.0(1.28, 3.28)	98.0		
20-24	0.9(0.88, 0.92)	99.9		
25-29	0.6(0.58, 0.62)	99.4		
30-34	0.6(0.57,0.62)	99.4		
35-39	1.1(0.49, 1.59)	98.9		
40-44	1.6(0.70,2.23)	98.4		
45-49	1.9(0.79, 2.69)	98.1		
**Education**			5.99	0.112
No education	1.0(0.17, 1.17)	99.0		
Primary	1.3(0.56,1.86)	98.7		
Secondary and higher	1.0(0.58, 1.58)	99.0		
**Wealth**			3.27	0.514
Poorest	1.3(0.44, 1.74)	98.7		
Poorer	0.9(0.89, 0.91)	99.1		
Middle	0.6(0.58, 0.62)	99.4		
Richer	1.6(0.62,2.22)	98.4		
Richest	1.0(0.45, 1.45)	99.0		
**Parity**			9.19	0.010
1-4	0.9(0.89, 0.91)	99.1		
5-9	1.1(0.29, 1.29)	98.9		
10+	2.4(1.44, 3.84)	97.6		
Age at first birth			2.71	0.260
<15	1.2(0.67, 1.87)	98.8		
15-20	0.98(0.97, 0.99)	99.02		
>20	0.9(0.88, 0.91)	99.1		
**Circumcision**			4.41	0.036
Yes	1.5(0.20, 1.70)	98.5		
No	1.0(0.39, 1.39)	99.0		
**Age at marriage**			0.89	0.640
<15	1.3(0.34, 1.64)	98.7		
15-20	0.8(0.79, 0.81)	99.2		
>20	0.9(0.88, 0.92)	99.1		
Height			3.99	0.046
<1.5 meter	0.2(0.17, 0.23)	99.8		
> = 1.5 meters	1.1(0.28, 1.38)	98.9		
Marriage by abduction			0.180	0.671
Yes	1.3(0.73, 2.03)	98.7		
No	1.1(0.18, 1.28)	98.9		

**Figure 1:**
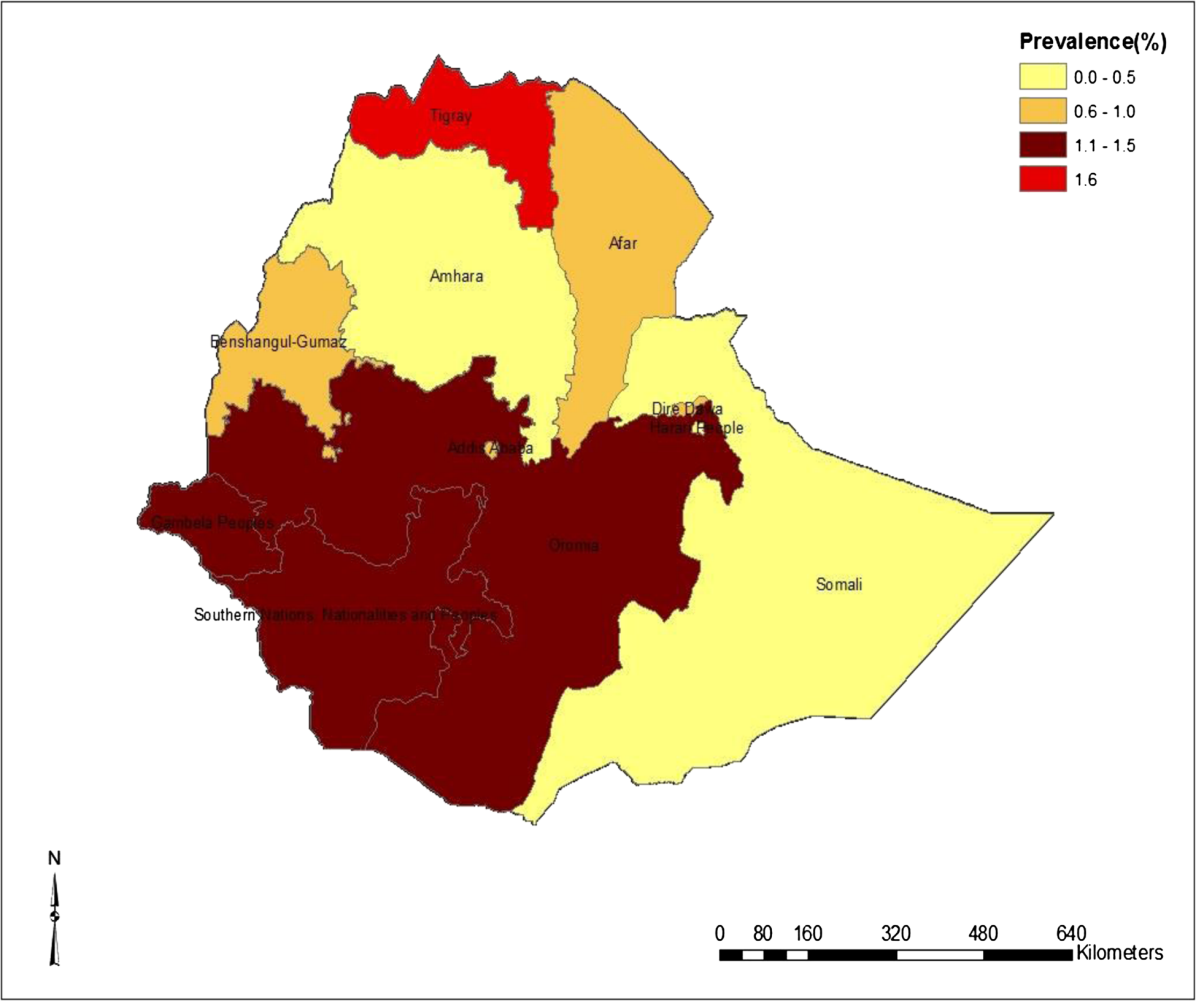
Prevalence of Obstetric Fistula among women who ever given birth in Ethiopia.

**Figure 2:**
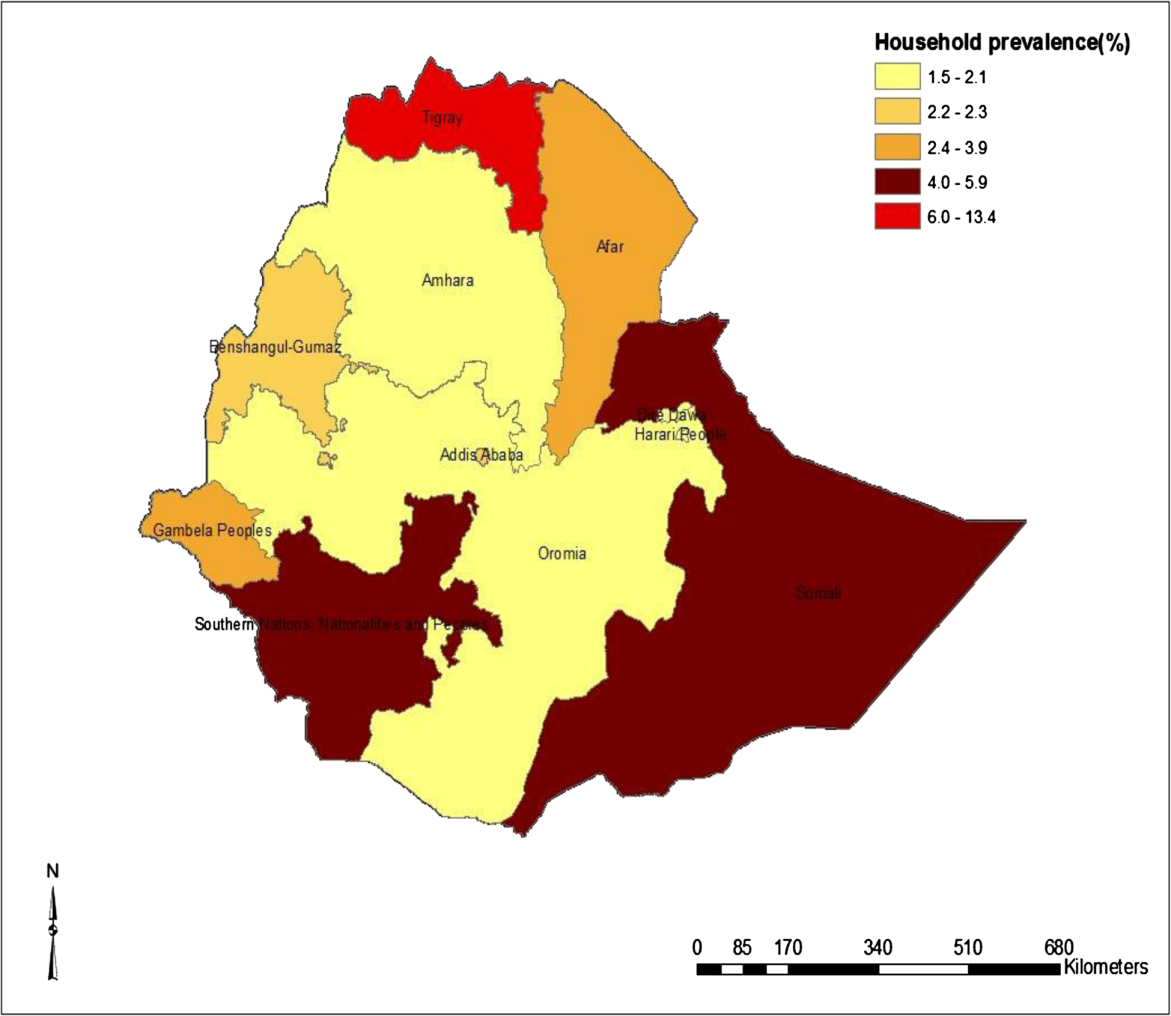
Percentage of other women in household with obstetric fistula.

The prevalence of obstetric fistula varies among the regions. Tigray (1.6%) and Southern Nations, Nationalities, and People’s Region (SNNPR) (1.5%) had high prevalence of OF followed by Oromiya (1.2%). No cases of OF was identified from the Somali region. Nonetheless 5.9% of women reported that they have OF cases in their households in Somali region. Similar to women’s own self-reported prevalence, Tigray region was recorded to have the highest household level prevalence of obstetric fistula, followed by Somali (5.9%) and SNNPR (5.0%). Figure 1 shows the map of prevalence of obstetric fistula in Ethiopia (women who reported to have obstetric fistula), whereas Figure 2 shows the map of women who reported to have OF cases in their households.

### Predictors of obstetric fistula

In cross tabulation women in the age group 15-19 and those in age 40 and above had higher odds of experiencing fistula than the other age groups (Chi square = 12.7, p = 0.048). Similarly those who are from Tigray and SNNPR regions had higher odds of obstetric fistula prevalence (Chi square = 26.6, p = 0.003). Those women who are circumcised had higher odds of occurrence of the condition (Chi square = 4.41, p-value = 0.036). Those women who delivered 10 or more times were more likely to report obstetric fistula (chi square = 9.19, p = 0.01). In the logistic regression model women from rural areas were less likely to report obstetric fistula than their urban counterparts (OR = 0.21, 95% CI: 0.06-0.69). Women who gave birth 10 or more times were more likely to develop obstetric fistula than women with 1-4 child (OR = 4.34; 95% CI; 1.29-14.55) (Table 2).

**Table 2:** Fistula by background characteristics using logistic regression

**Variable**	**Fistula**		**Unadjusted OR**	**Adjusted OR£**
			**95% CI**	**95%CI**
	**Yes (%)**	**No (%)**		
**Residence**				
Urban	1.4	98.6	1	1
Rural	1.0	99.0	0.72(0.47–1.10)	0.21(0.01–0.06)
Parity				
1-4	0.9	99.1	1	1
5-9	1.1	98.9	1.22(0.79–1.89)	0.68(0.27–1.70)
10+	2.4	97.6	2.80(1.40–5.58)	4.34(1.29–14.55)

£ Adjusted for socio demographic characteristics (age, height, education, wealth, and region) reproductive health characteristics (parity, age at first marriage, age at first birth, circumcision).

## Discussion

**Figure 3:**
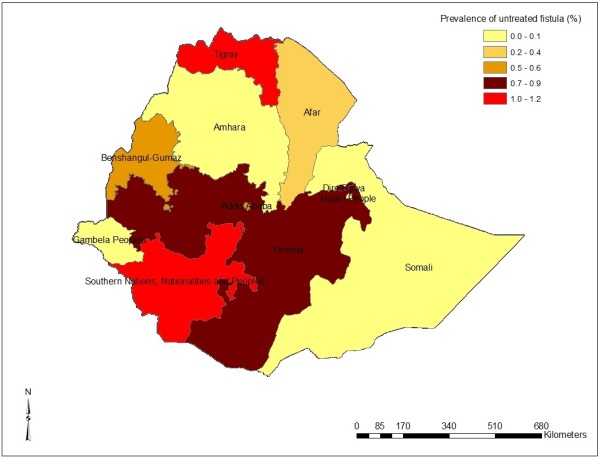
Regional distribution of untreated fistula in Ethiopia.

The findings indicated that the prevalence of OF was 10.6 per 1000 women who ever gave birth. Majority (68.0%) of the identified cases were not treated at the time of the survey indicating the prevalence of untreated fistula to be 7.2 per 1000 women who ever gave birth (Figure 3). There were significant regional variation in the prevalence of OF in the country, high prevalence was registered in Tigray and SNNPR. The analysis indicated women from urban area and women who delivered more than 10 times were more likely to experience OF. Nonetheless the measurement used to diagnosis OF in our study was questionnaire based, which might overestimates the findings as compared to other studies which used clinical diagnosis.

The time and measurements of OF in different studies show variation making it difficult to make comparison. In our study the prevalence of OF was (103/14070) 7.3 per 1000 women aged 15 to 49 years, which is higher than 2.2 per 1000 of women between 15 and 49 years of age reported in rural Ethiopia [9], 1.88 per 1000 women aged 15 to 49 years in South Saharan Africa [8] and 2 to 3 per 1000 in Nigeria [6] and is lower than the 26 per 1000 women in reproductive age reported in Uganda [10]. Based on a prevalence of 7.3 per 1000 (95% CI: 5.9-8.7) women aged 15 to 49 years it is estimated that in Ethiopia 142,387 (95% CI: 115080, 169694) OF patients exist. This highlights the number of women living with OF is substantially higher than the previous estimates. The difference between our estimation and Muleta *et al*[10] could be different in the measurements. In our study OF was measured using questionnaire which entirely dependent on the understanding of the interviewed women. In the other study physical examination by physician was conducted. Obstetric fistula is a double concern in that most (68.0%) of the OF cases identified in the study are untreated. Although OF treatments services are available in Ethiopia, the results here indicate that they are far from reaching the treatment needs in the community. Community based initiative to identify cases of OF and linking them with the treatment providing facilities are important to reduce the cases of untreated OF patients in the country.

The study indicated the presence of geographical variation in the prevalence of OF in the country. Tigray and SNNPR are the two regions with high prevalence of OF. To identify the reasons behind the regional variation there needs to be detailed studies examining multiple variables such as culture, utilization of health services, health awareness etc. However in previous years it was assumed that Amhara region was indicated to be the region with high prevalence of OF, the community based education program offered by many organizations and fistula campaigns targeting the region might have implicated in the reduction of the number of cases of OF in the region. The result here warrants the establishment of OF treatment centers in these regions to address the needs of OF cases, in addition to the continued efforts in emergency obstetric care services and reinforcing the lunched Campaign to End Fistula. The Campaign is devoted to galvanizing commitment and resources for the elimination of obstetric fistula as well as drawing attention to pernicious gaps in maternal health services particularly for the poor and to gender inequities that limit women’s prospects and autonomy. Inspiration for the Campaign came from the Addis Ababa Fistula Hospital which has been providing holistic treatment for women suffering from fistula for over thirty years [11]. The campaign was initiated from the interest of Ministry of Health of Ethiopia, the Hamlin Fistula hospital, and USAID [12].

One of the factors for experiencing OF was residence. Studies demonstrated that major risk factors for the development of a fistula included living in rural residence [13-15]. However in our study it is demonstrated that cases were found to be prevalent in urban settings than rural. These discrepancies could be due to differences in measurements and time variants. In our study current place of residence was recorded rather than the place of residence during the index pregnancy leading to OF and in most of the cases the OF patients had gone to the urban areas for care and treatment. This is evidenced and an established report concludes that OF more commonly comes from rural areas where there is a lack of obstetric services, and rarely from urban areas. Women with fistulas are no longer able to successfully fulfill their societal role of wife and mother, and are often deserted by husbands and family and stigmatized by society [16].OF is stigmatizing disease in Ethiopia and patients usually tend to migrate to urban areas in fear of stigma and discrimination. In addition most of the OF treatment centers are found in big cities and most of the patients coming for treatment tend to stay in the urban settings after treatment. Even to the level that the reduction of stigma remains a major challenge for public health programs involved with the problem of fistula [17] and virtually every study of obstetric fistula mentions that the social stigma associated with this condition and states that women with fistulas are often ostracized [18].

In our study, as the number of pregnancy increase the likelihood of fistula development increase gradually. In contrast in Ethiopia, studies show that primiparity had the strongest and most consistent association with longer duration of labor, urethral damage and vaginal scarring or obliteration [19]. Obstetric fistula is more commonly associated with primiparous mothers. Primipara have a longer and more damaging labor [20] and women living with fistula were found to be, for the most part, primiparous from previous studies [6,21-23]. This might have a programmatic implication for focusing the increased frequency of pregnancy might end up with pelvic wall inability to hold the pregnancy and this is also supported by different literatures as higher parity is associated with increased likelihood of incontinence from basic pelvic floor weakness [24]. Interestingly, nearly 25% of the patients had a parity of 5 or more, indicating that labor can become obstructed even in women who have previously delivered vaginally. This probably represents the tendency for birth weights to increase with successive pregnancies, as well as the effects of aging on changes in pelvic anatomy [25]. Similar to our finding there were a report of multiparous women, higher incidence of vesico-vaginal fistula [26]. In addition in our study the current party was measured, which might not indicate the number of pregnancy during the index pregnancy of OF.

Some limitations were observed in the interpretation finding of this study. The questionnaire probably including many cases of urinary incontinence from other sources (stress urinary incontinence, detrusor overactivity, mixed incontinence, overflow) unlike to other study whose only included cases confirmed positive by examination, excluding many who screened positive on initial questioning) that might inflate the prevalence and the occurrence of Obstetric fistula is a clinical diagnosis that is not diagnosable by a questionnaire base. Even Measuring obstetric fistula at the population level has its own drawback in which Obstetric fistula is believed to be a statistically rare event, and therefore a large sample size is required [18].

## Conclusion and recommendations

The study demonstrated that the prevalence of fistula in Ethiopia is high as compared to other developing countries. Regional differences were observed Tigray and SNNPR reporting high prevalence of OF. Parity and living in urban area were also the determinate factor for the presence of fistula. Therefore prevention of OF and intensifying case finding to end the suffering OF cases is recommended in addition to accelerating the expansion of emergency obstetric care services.

## Competing interests

The authors declare that they have no competing interests.

## Authors’ contributions

SB and YL conceived the study and involved in data analysis, and drafted the manuscript and reviewed the article. AAR was involved in the data analysis and drafted and reviewed the article. KD participated in the data analysis, produced the GIS maps and contributed to report writing and drafted and reviewed the article. All authors read and approved the final manuscript.
